# Access to health services for men who have sex with men and transgender women in Beira, Mozambique: A qualitative study

**DOI:** 10.1371/journal.pone.0228307

**Published:** 2020-01-30

**Authors:** Farisai Gamariel, Petros Isaakidis, Ivan Alejandro Pulido Tarquino, José Carlos Beirão, Lucy O’Connell, Nordino Mulieca, Heitor Pedro Gatoma, Vasco Francisco Japissane Cumbe, Emilie Venables

**Affiliations:** 1 Médecins Sans Frontières, Beira, Mozambique; 2 Médecins Sans Frontières, Southern Africa Medical Unit (SAMU), Cape Town, South Africa; 3 Médecins Sans Frontières, Maputo, Mozambique; 4 Ministry of Health, Beira, Mozambique; 5 Division of Social and Behavioural Sciences, School of Public Health and Family Medicine,University of Cape Town, Cape Town, South Africa; University of South Florida, UNITED STATES

## Abstract

**Objectives:**

HIV prevalence and incidence are higher among key populations including Men who have Sex with Men (MSM) and transgender women in low and middle income countries, when compared to the general population. Despite World Health Organisation guidelines on the provision of services to key populations recommending an evidence-based, culturally relevant and rights-based approach, uptake of HIV services in many resource-limited and rights-constrained settings remains low. Médecins Sans Frontières (MSF) has been offering health services for MSM and transgender women in Beira, Mozambique since 2014 using a peer-educator driven model, but uptake of services has not been as high as expected. This qualitative study aimed to learn more about these key populations in Beira, their experiences of accessing MSM- and transgender-friendly services and their use of face-to-face and virtual networks, including social media, for engagement with health care.

**Methods:**

In-depth interviews were carried out with MSM and transgender women who were 1) enrolled in, 2) disengaged from or 3) never engaged in MSF’s programme. Purposive and snowball sampling were used to recruit the different groups of interviewees. Interviews were conducted in Portuguese, transcribed and translated into English before being coded and manually analysed using a thematic network framework.

**Results:**

Nine transgender women and 18 cisgender MSM participated in the study. Interviewees ranged in age from 19 to 47 years, with a median age of 29. Three main themes emerged from the data: perceptions of stigma and discrimination, experiences of the peer-educator driven model and the use of face-to-face and virtual platforms for communication and engagement, including social media. Interviewees reported experiencing stigma and discrimination because of their gender or sexual identity. HIV-related stigma and health-care setting discrimination, including gossip and breach of confidentiality, were also reported. Although the presence of the peer-educators and their outreach activities were appreciated, they had limited visibility and an over-focus on health and HIV. The face-to-face networks of MSM and transgender women were small and fragmented. Virtual networks such as Facebook were mainly used for flirting, dating and informal communication. Most interviewees were at ease using social media and would consider it as a means of engaging with health messaging.

**Conclusions:**

MSM and transgender women have challenges in accessing health services due to being stigmatised because of their gender identity and their sexual behaviour, and often experience stigma at home, in health-care facilities and in their communities. Peer-driven models of engagement were appreciated but have limitations. There is an untapped potential for further expansion and engagement with face-to-face and virtual platforms to reach MSM and transgender women in settings with a high HIV burden, and to provide them with essential information about HIV and their health.

## Introduction

HIV prevalence and incidence are higher among men having sex with men (MSM) and transgender women in low and middle income countries when compared to men in the general population [1–3]. Despite World Health Organization (WHO) guidelines on the provision of services to key populations including MSM and transgender women which recommend an evidence-based, culturally relevant and rights-based approach, uptake of HIV services in many resource-limited and rights-constrained settings remains low [2]. The guidelines stipulate that there should be provision of a comprehensive package based on evidence of HIV-related recommendations, increased awareness of needs and important issues, improved access, coverage, uptake and acceptable health services and national and global funding for these services (WHO, 2014). Local health sectors and key partners such as Non Governmental Organisations (NGOs) are typically responsible for the provision of these services.

Stigma and discrimination can hamper progress in working with key populations in many different contexts [4–5]. Homosexuality has been decriminalized in Mozambique since June 2015 but MSM and transgender women still experience stigma and discrimination in their daily lives and when accessing health-care services, often linked to social, cultural and religious norms. Although it is against the law in Mozambique to discriminate against an individual based on their gender, more needs to be done by the government to safeguard the interests of this group. The government is being urged to do more to curb exclusion and marginalization of the LGBT community in Mozambique [6].

As such, MSM and transgender women remain extremely vulnerable to adverse health outcomes due to fear of stigma and discrimination while seeking or receiving care and services. Perceived and anticipated stigmatisation against homosexuals has been reported in Malawi, Namibia, Zambia, Zimbabwe, as well as in Mozambique [7]. Although MSM-friendly, tailor-made health services such as clinics for men, door-to-door follow up from peers and after hour clinics are offered, MSM still encounter barriers in access [8]. According to the United Nations Programme on AIDS (UNAIDS), discrimination in health care settings reduces the quality of life of individuals who experience it [9].

In January 2016 Médecins Sans Frontières (MSF) introduced a peer-educator led outreach model of care providing home visits, HIV and Sexually Transmitted Infection (STI) testing and screening, access to antiretroviral treatment and psycho-social support to MSM and transgender women in Beira, Mozambique. These services were introduced to provide friendly, personalised services in a safe, confidential space distinct from mainstream health-care facilities. Despite progress made since the model was implemented, challenges in engaging with and recruiting new beneficiaries into services remain, and alternative strategies are required.

As an alternative to face-to-face engagement with other peers, social media platforms may enable key populations who are fearful or reluctant to be seen accessing health services or asking for information on HIV prevention and treatment. Social media can be used in different social, economic and cultural contexts, with messages being used for communicating information relating to HIV prevention and treatment [10]. Social media may also offer anonymity and confidentiality to users, which face to face interaction does not [11].

This study explores MSM and transgender women’s experiences of accessing health-care services in Beira, Mozambique; discusses their knowledge and experiences of a peer-based outreach model of care and explores face-to-face and virtual strategies for engagement that could ultimately improve access to care.

## Methods

### Study design

This was an exploratory qualitative study involving in-depth interviews with MSM and transgender women in Beira, Mozambique. Data collection took place between July and September 2018.

### Programme description

MSF has been working in Beira since June 2014, when a project offering HIV prevention and treatment, including sexual and reproductive health (SRH) services, to female sex-workers was first implemented. The programme was gradually expanded to other key populations and the first MSM beneficiary was registered in January 2016.

A clinic for men, HIV testing and door-to-door follow up services as well as Pre-Exposure Prophylaxis (PrEP) for HIV negative beneficiaries are provided in the project, along with community outreach activities for key populations. Community outreach activities include mobilization and sensitization of MSM and transgender women to assist with accessing HIV counselling, testing and care and STI screening and treatment. Condoms, lubricant and educational materials are also distributed to beneficiaries.

A peer driven model of care forms the basis of the outreach activities of the project. Members of MSM and transgender communities in Beira are recruited and trained in engaging with their communities to promote utilization of existing health services. Peer educators are also trained to perform tasks that would otherwise have been carried out at a clinic by doctors and nurses, such as counselling, testing and referrals of individuals. However, the main aim of this model was not to support task shifting to lay cadres as much as it was to provide access to services for marginalized and isolated individual and groups using a peer-based approach.

Despite the introduction of these services and the involvement of MSM and transgender women peer-educators in the project, uptake of services amongst MSM and transgender women remains low. The current beneficiaries of the project are mainly young adults aged 18 to 30 years of age who are either attending school or looking for employment. The estimated population of MSM in the city of Beira is 2,600 [12], and by end of November 2018, approximately 557 MSM were enrolled in MSF’s programme. Figures for transgender women were not available.

### Study population

Interviews were conducted with those who defined as MSM (that is, cisgender men who have sex with other cisgender men and cisgender or transgender women) and transgender women (participants who identify themselves as women but were assigned male sex at birth). We did not ask detailed questions about who interviewees had sexual relationships with (e.g. if a transgender woman had relationships with MSM and cismen) as this was not the purpose of the study. Three different categories of MSM and transgender women were included in the study; those who are currently enrolled in MSF’s programme, those who have disengaged from the programme and are no longer considered ‘active’ beneficiaries, and those who have never enrolled in the programme. We have included these three categories of participants and we present the quotations using three identifiers: Active In Care (AIC), disengaged from care (DFC) and never engaged in care (NE).

Participants who were still considered to be active in care were selected to explore their perceptions on the current package, including peer-driven care and outreach, being offered to MSM and transgender women. Those who had disengaged from care were included to explore their reasons for no longer accessing services and to explore possible solutions to prevent others from dropping out of care in the future. Participants who had never engaged with MSF’s services were included in the study to understand their reasons for non-engagement of the services being offered to them.

### Recruitment and sampling

Purposive sampling was used to select participants who were actively enrolled in the programme, as well as those who had disengaged from care. Their phone numbers were retrieved from the MSF database by the Principal Investigator (PI) who then telephoned them to explain the study and ask if they would be willing to participate. Those who did not have telephone numbers available were contacted at home by a trained peer-educator and informed about the study.

MSM and transgender women who had never joined MSF’s programme were identified in their local communities by peer-educators and invited to participate in the study. Snowball sampling was then used to identify potential interviewees through the networks of those who had already been interviewed. Participants who were not enrolled in the programme were asked to identify other participants in their network to assist the research team with reaching those who were ‘hidden’ or ‘hard to reach’. The participants were asked to consider if there was anyone else (MSM or transgender women) in their networks who they thought would be interested in participating in the study and inform them about the research. If they agreed, their contact details were then passed on to the PI for recruitment purposes.

Potential participants were told that the study was being conducted to learn more about MSM and transgender women in Beira, their experiences and challenges of accessing health-care services and their recommendations for how MSF could improve their services in future. Some participants were familiar with the PI as they were from the same community (and were also told that the PI had an interest in the study topic because of their work with MSF), but all participants were reassured that their participation and responses would be anonymized and not shared with others.

### Data collection

All in-depth interviews were carried out in Portuguese by the PI, as this was the language spoken by all of the interviewees and is a language in which the PI is fluent. The PI has an MSc and training in qualitative research methods, and at the time of the study was working as an operational research assistant in the project. Interviews were conducted using guides which had been pre-tested with MSF peer-educators. Interview guides contained open-ended questions and the PI probed for further information during interviews. Interview guides contained different questions depending on whether or not the participant was enrolled in MSF’s programme, had disengaged from care or had never been enrolled (S1 File). During interviews, all participants were asked to discuss their knowledge of MSF’s services and their use of social media and other social networks.

The interviews were conducted in interviewees’ homes, *barracas* (small informal bars), barber shops, in *tuktuks*, isolated areas under trees and in the MSF office depending on the preference of the interviewee. Locations were chosen to ensure privacy and to reduce any risk of accidental disclosure. Only the PI and the interviewee were present during the IDIs. One participant dropped out of the study as they did not want to continue the interview once they understood more about the content of the questions. Each interviewee was interviewed once: no repeat interviews were conducted. Interviews ranged in duration from 17 to 36 minutes, with a median duration of 22 minutes. Saturation was not reached in all categories as it was extremely difficult to find and recruit participants who were no longer engaged in care.

### Audio-recording, transcription and translation

Interviews were audio-recorded and handwritten notes were also taken by the PI to record any non-verbal communication or other points of interest. All audio-recorded interviews were transcribed into Portuguese before being translated into English. Transcriptions were carried out by three members of the research team and translation from Portuguese to English was conducted by the PI. The two co-investigators who transcribed the interviews were asked to verify any discrepancies that emerged during transcription.

### Data analysis

Transcripts were manually coded by the PI with support from three co-investigators. An inductive approach to coding was applied. A thematic network approach, as described by Attride-Stirling, was used, where large text was decoded into smaller but meaningful data that could be easily interpreted for analysis [13]. Basic themes were grouped together with their categories and sub themes and then grouped under larger organizing themes around the global theme of access to health services. Results were presented to the MSF project team for feedback, but we were unable to share transcripts with interviewees for further review or discussion. The three main themes and their corresponding subthemes are presented below.

This study is reported in accordance to the Consolidated Criteria for Reporting Qualitative Research (COREQ) checklist [14] (S2 File).

### Ethics approval

The study protocol (S1 File) was approved by MSF Ethics Review Board, Geneva, Switzerland (ID 1830) and the Mozambican *Comité Nacional de Bioética para Saúde* in Maputo, Mozambique (186/CNBS/18). Informed consent, including specific consent for the use of quotations in any future publication, was obtained from all study participants before interviews began.

## Results

A total of 27 in-depth interviews were conducted: nine transgender women and 18 MSM participated in the study.

The characteristics of the interviewees as they were reported to the PI are shown in Table 1. Three main themes relating to the use of health services by MSM and transgender women emerged from the data: 1) perceptions of stigma and discrimination, 2) experiences of a peer-driven outreach model for HIV service provision and 3) the use of virtual and face-to-face platforms for engagement with such groups.

**Table 1:** Interviewee category.

Interviewee code	Age range	Gender	Sexuality
NE001*	40–45	Transgender woman	Has sex with men
**NE002**	20–25	Cisgender man	MSM
**NE003**	30–35	Transgender woman	Has sex with men
**NE004**	45–50	Cisgender man	MSM
**NE005**	30–35	Cisgender man	MSM
**NE006**	30–35	Cisgender man	MSM
**NE007**	45–50	Cisgender man	MSM
**NE008**	40–45	Cisgender man	MSM
**NE009**	40–45	Cisgender man	MSM
**NE010**	25–30	Cisgender man	MSM
**NE011**	25–30	Cisgender man	MSM
**NE012**	25–30	Cisgender man	MSM
**NE013**	20–25	Cisgender man	MSM
**NE014**	30–35	Cisgender man	MSM
**NE015**	20–25	Transgender woman	Has sex with men
DFC001**	25–30	Cisgender man	MSM
**DFC002**	40–45	Cisgender man	MSM
AIC001***	25–30	Cisgender man	MSM
**AIC002**	35–40	Transgender woman	Has sex with men
**AIC003**	20–25	Transgender woman	Has sex with men
**AIC004**	25–30	Cisgender man	MSM
**AIC005**	20–25	Cisgender man	MSM
**AIC006**	<20	Transgender woman	Has sex with men
**AIC007**	25–30	Cisgender man	MSM
**AIC008**	20–25	Transgender woman	Has sex with men
**AIC009**	20–25	Transgender woman	Has sex with men
**AIC010**	25–30	Transgender woman	Has sex with men

*Active In Care **(AIC)** **Disengaged From Care **(DFC)** ***Never Engaged in Care **(NE); MSM;** men who have sex with men

### Stigma and discrimination

Many interviewees described feeling stigmatised and discriminated against in the wider community and in health-care facilities because they self-identified as MSM or transgender. The sub-themes presented in this section include MSM and transgender-related discrimination; gossip and lack of confidentiality; HIV-related discrimination and health-care level discrimination.

### MSM and transgender-related discrimination

Most out of interviewees described feeling ‘hidden’ and marginalised because of their sexual or gender identity, as the example below demonstrates. Interviewees were reluctant to be publicly open about being MSM or transgender for fear of rejection and were not comfortable disclosing their sexuality or gender identity with others:

…not everyone has come out of the closet and is open. There are some who are still inside the closet, there are people who, because of their social, religious, and family position, end up staying [there]. (DFC, Transgender woman, 25–30 years)

This male interviewee felt that he needed to keep his relationships with other men hidden for fear of societal disapproval:

So when you go out with a man, not everyone has to know about this. Society does not look at these things [homosexuality] with good eyes. (NE, MSM, 30–35 y)

### Gossip and lack of confidentiality

Interviewees, including the MSM and transgender woman cited below, were concerned about gossip and the potential breach of confidentiality by health care providers, which in turn created a reluctance to access services.

Nowadays, there are no friends. You can say "*this here is my friend*", talk to him, when there is a bomb (a secret), you end up arguing, and then he takes that bomb, talks to someone else. I do not trust people so much. No, I do not. (AIC, MSM, 20–25 y)

A couple of peer-educators there…speak a lot. For example, they go to the field. They will do an [HIV] test of a person or a beneficiary, they will do a test. Then that person arrives, he talks with his colleagues. (NE, Trans woman, 40–45 y)

### HIV-related discrimination

Interviewees highlighted that HIV-related discrimination was very common for them. Discrimination was linked to being HIV positive as well as to being seen accessing HIV prevention services.

The peer educator himself, in addition to calling the beneficiary to a closed site on the street itself shouted to their colleagues that the beneficiary still coming. Why didn’t you come and do CD4? (NE, Trans woman, 40–45 y)

Interviewees were afraid to disclose their HIV status for fear of stigma and discrimination and did not want other people to discuss their status or testing. They were particularly worried about people disclosing their HIV status to others without their consent as this could lead to ‘defamation’, as described below:

I'm worried. Why did you do this [disclose] without my permission? This is defamation. (NE, MSM, 20–25 y)

### Health-care level discrimination

Experiencing stigma and discrimination in health care settings was viewed by interviewees as a deterrent from seeking services. The participant below was one of several people who described a discriminatory incident that occurred when seeking health care.

They were barred by discrimination even before entering through the door, but before being in there…outside, you see? They were discriminated against. I usually hear the complaints after they go out there, saying “*I'll never put my feet in there again because I was discriminated against*”. A stare like this, and other people laughing. (NE, Trans woman, 40–45 y)

Another interviewee reported concerns and reluctance in accessing health-care as a transgender woman because she feared being stigmatised against:

One day I was ill with problems in the anus. I went and received treatment [from the health centre], they helped me a lot. I could not…I went to the hospital several times and I could not [be treated], they gave me pills and it did not work. I was ashamed. (AIC, Trans woman, 20–25 y)

## Peer-driven outreach model of service provision

The following results relate to participant perceptions and experiences of the peer-driven model of service provision described above, including the visibility of the model; perceived benefits of the model; the provision of condoms and lubricant; relationships with peer-educators and challenges to the model.

### Visibility of the model and services

Many interviewees who had not enrolled in the programme were not aware of the specific services offered by MSF to MSM and transgender women. When asked about the visibility of MSF’s services, it was clear that not all interviewees had heard of the programme or met the peer educators:

I've never heard of it. I've heard that there is a clinic for men, but I did not know that it was particularly for MSM. (NE, MSM, 30–35 y)

I have never heard [of these services]. No-one from MSF has ever come to me to talk about their services. (NE, MSM, 30–35 y)

There was also a lack of awareness of MSF’s MSM and transgender friendly services amongst interviewees from more remote neighbourhoods of Beira. Interviewees, such as the man cited below, from these neighbourhoods could not recall any leaflets, banners or other informational material being displayed or handed out.

No, it is the first time to hear it. I have never had contact with any peer educator. (NE, MSM, 30–35 y)

### Perceived benefits of the model

Many of the interviewees who had experience of MSF’s programme talked about the benefits of peer support and the role of peer-educators in providing health-care services for MSM and transgender groups in Beira. There were, however, other interviewees who described their hesitancy in enrolling in the programme.

I liked the service, the advice, and I saw that this will be a benefit for me and for my health. At any moment they [peer educators] support me. (AIC, MSM, 20–25 y)

There are some people who do not have courage, they are ashamed. I do not know why. I usually invite many friends to my house, and when they come to my house I give them a hand [support]. Some are following and some are not following [my advice], but I have nothing to do with this. I only know about the ones who are following it… (AIC, Transgender woman, 35–40 y)

I know the benefits of being there [active in care] because if I am enrolled I can help others through talking to them (NE, MSM, 30–35 y)

Home visits were highlighted by the participants who were enrolled in the programme as an important element of the personalised services provided. They were particularly appreciative of this service as it meant they did not have to go a health-care centre or disrupt their daily routine to access services.

### Provision of condoms and lubricant

Those who had received MSF’s services appreciated having condoms and lubricant delivered to them at home, and also appreciated the counselling and information that they were given. Some participants highlighted that before MSF’s services began they had difficulties in finding condoms in Beira, and that because lubricant was not readily or freely available they had to use saliva or local herbs instead.

Lubricating gel, condoms…if everything has finished I call them [peer educators] and they come and give me straight away at home. It is not necessary for me to spend transport fare to go there. (AIC, Trans woman, 37–40 y)

I always get condoms, lubricating gel; I am already counselled (NE, MSM, 45–50 y)

### Relationships with peer-educators

MSM and transgender women who are engaged with MSF’s services often maintain close contact with peer educators, which enables them to seek advice and information when needed.

If I do not have it, I always call my [peer educator] and say that I "*no longer have condoms or lubricant*, *I want to test*" and he always comes to me and provides me with the services. (AIC, MSM, 25–30 y)

The peer educators provide me with services. I have good communication with them and they have good communication with me. So we have good communication between the two of us. (AIC, MSM, 25–30 y)

### Perceived challenges of the model

There were several reported challenges with the peer-educator model, which are related to a lack of regular follow up and a lack of time for engagement from enrolled participants, as well as concerns about confidentiality. The extremely positive perceptions of peer-support and personalized service delivery were challenged by the lack of systematic or consistent follow up.

I only know that they came that day. This person was giving a health talk, everything, then never came anymore. (DFC, MSM, 40–45 y)

Some interviewees had not been visited by the peer educators since initial contact was made. One interviewee who had been enrolled in the MSF programme before disengaging from care stated that;

They should contact you frequently; they should not get in touch with you every five years, as then that person ends up being off your list. (DFC, MSM, 25–30 y)

### Breach of confidentiality by peer educators

Interviewees who had been part of MSF’s programme cited challenges with confidentiality as one of the main problems of the model.

A couple of peer-educators there…speak a lot. For example, they go to the field. They will do an [HIV] test of a person or a beneficiary, they will do a test. Then that person arrives, he talks with his colleagues. […] The peer educator himself, in addition to calling the beneficiary to a closed site on the street itself shouted to their colleagues that the beneficiary still coming. Why didn’t you come and do CD4? (NE, Trans woman, 40–45 y)

## Expansion of virtual and face-to-face networks

Interviewees discussed the physical places in which they socialized and met other MSM and transgender women, as well as talking about the social media platforms that they used to talk to other people. We now present the results relating to the use of face-to-face networks, virtual networks and social media.

### Face-to-face networks

Interviewees described the different places they gathered to socialize and meet sexual partners. Many met in specific places such as *barracas* (small, informal bars), barber-shops and in their friends’ or sexual partners’ homes, as these were places where they felt safe. Some also described participating in social gatherings in people’s homes or *barracas* which were not in the centre of town or in public places and thus less likely to draw attention to them.

Partners, sometimes they call to meet us here at home, sometimes we find ourselves in those places [*barracas*] but not for serious commitment with them. It happens a lot here at home, you see? (DFC, MSM, 40–45 y)

Nearby there is a barber shop there. There's a *mana* [transgender woman], but she is fake [she pretends she is not transgender], except … The boys all from here go there and stay focused [dating her]. They are really concentrated. (NE, Transwoman, 40–45 y)

MSM and transgender interviewees described how they met other people, including sexual partners, as well as how they developed their social networks. They explained how people met in small groups, often to avoid being stigmatized by others. One participant likeneda soccer event in their community to ‘mini LAMBDA’ (a local Mozambican NGO advocating for LBGT rights).

I have a group from the zone [neighbourhood], I have a soccer game group, I have a work group, I have a group of friends, a mini LAMBDA. ]. (NE, MSM, 30–35 y)

First I'll tell you that here in Beira, there are many gays. But many are still hidden. They hide for fear of reprisal. So it happens that there are a very small number of friends who come together and talk about it. But we will never find a large group because many are ashamed or afraid. […] I believe that if we had a niche, an office or a place that we could meet and get together and talk in an enclosed space, as time goes on, people would get used to it. (NE, MSM, 40–45 y)

Participants highlighted that it is rare to find a large group of MSM or transgender women together during social occasions because many people do not feel comfortable talking to others about their sexuality.

### Virtual networks and social media

The majority of the interviewees had smartphones and used social media networks including Facebook, WhatsApp and Instagram. Most of the participants used their smartphones to communicate with others for networking, flirting and dating. These forms of communication were used as interviewees did not ‘*always have time to be together*’.

Yes, Facebook is the platform that is really at the peak, then Twitter and WhatsApp. But what is really at the top is Facebook. (NE, MSM, 30–35 y)

Of course we use Facebook. From Facebook we go down [to find] the contacts [then] we go to WhatsApp. (NE, MSM, 20–25 y)

Health related matters were not commonly discussed on social networks, as these were mostly used for socializing and communicating with friends or sexual partners.

We do not talk much about health. We start talking now… (AIC, MSM, 20–25 y)

On social networks there is very little [about health]. Because people are reserved, they do not talk much. (NE, MSM, 40–45 y)

Whilst health related issues were not commonly addressed using social media, interviewees recognized that sending information could be a useful way of communicating about them. Many participants welcomed the idea of using such messages to communicate and share information about their health, particularly HIV.

I would like it because it would explain something to me that I did not understand. If they send me a message I would get to know that I have to take care of myself more, that I have to protect myself more to avoid some diseases. (NE, MSM, 25–30 y)

If I got messages about health, I would feel very comfortable. (NE, Transgender woman, 30–35 y)

For some interviewees, virtual platforms such as Facebook or WhatsApp were considered important ways to help them to feel part of a wider community and receive information not only about health, but about their rights.

It would feel good. I would feel really good. You see a community where you are … a community that you attend that also puts something on Facebook talking about health, about the right of homosexuals…it would be very good. It is important. It's good. It's good. (AIC, MSM, 25–30 y)

Now, if you join a network, an organization where you will give content on how I can be healthy, how [health] can be addressed, that already reassures me. (NE, MSM, 40–45 y)

Interviewees suggested several practical ways in which MSF could further engage with MSM and transgender communities in Beira. One suggestion was to identify focal points in remote areas who could help to disseminate information about specific services for men in the area.

Several of the interviewees highlighted the need to talk about MSF in communities who were not aware of the services being provided:

Yes, I want you to spread the word to the most [at risk] people, such as those who are still in the closet, but also that MSF, work will be expanded even in the remote zones that they arrive until there, because there are bisexual people in remote areas too… (NE, MSM, 30–35 y)

A lot of lecturing is needed, because many do not know what MSF is. Talks in neighbourhoods, health centres so the population gets to know what MSF is. (NE, MSM, 25–30 y)

Interviewees also suggested working in local schools (where MSF is not currently present) to ‘*spread the information*.’

If they could expand and search for [peer educators] who could be available twenty-four hours a day, campaign in the remote areas and go to schools, that could help a lot. I’ve never had anyone from MSF ever came to me to talk about their services. (NE, MSM, 30–35 y)

## Discussion

This qualitative study has elicited some important findings about MSM and transgender communities in Beira, a semi-urban setting in Mozambique greatly affected by HIV and characterized by considerable stigma and discrimination towards key populations. We found that a peer-driven model was relatively well accepted by these communities, but its visibility and ability to integrate into multiple, closed and hidden networks of potential beneficiaries were limited. Expansion of face-to-face and virtual networks and platforms, especially the utilization of social media, may be key in unlocking the peer-driven model’s untapped potential in reaching increasing numbers of vulnerable people and expanding access to health messaging and services.

According to Wirtz et al., less than 10% of MSM globally are accessing MSM-specific services, and this is even lower in low and middle-income countries [15]. This is particularly worrying as several countries, including Uganda and Gambia, still criminalize homosexuality which in turn may further fuel the HIV epidemic [16]. Although homosexuality is no longer criminalized in Mozambique, local social and cultural norms still marginalise MSM and transgender women on the basis of their gender and their sexual behaviour. As described above, several of the interviewees in our study reported that they had experienced some form of discrimination either generally in their communities or when accessing health services. The fear of being stigmatised or having their sexual orientation or HIV status disclosed was a deterrent to using health and other support services, further adding to their vulnerabilities. These findings confirm findings from previous quantitative and qualitative studies, including those from the neighbouring country of South Africa [17]. A study conducted among gay men who visited clinics in Soweto showed that all the participants had experienced some form of discrimination, including being ridiculed or called names [18]. Similarly, in another study from South Africa, two out of three transgender participants reported negative experiences when visiting public clinics [19].

A recent study conductedin Senegal revealed that even though the country has managed to control HIV with a prevalence of 0.5% among adults 15–49 years, the situation was different among MSM, in which study participants reiterated that stigma was the main deterrent to accessing health services [20]. Even in a country such as South Africa where the government guarantees sexual and gender minorities the right to a non-discriminatory health-care system, homophobia and transphobia still exist and many believe that homosexuality is not part of African tradition and culture [17].

Peer-educators are often engaged in HIV programmes, particularly in the provision of community outreach services, as a way of supporting marginalized populations and providing services to those who may have difficulty in accessing them. Peer-led models of care have been proven to increase uptake of HIV related services to MSM [21].

Research on the use of social media or virtual platforms for engaging MSM and transgender populations has not previously been conducted in Mozambique, and in this first qualitative study targeting MSM and transgender women, most participants found the idea of using social media for health messaging beneficial and acceptable. Several public health interventions have incorporated social media in their engagement with MSM groups to increase access to health services [22, 23]. A study carried out among Afro-American and Latino MSM living in Los Angeles on the use of social media for communication in health, specifically on home-based HIV testing, concluded that social networking was acceptable to this population [22]. A further study conducted in China demonstrated behaviour change as a result of health promotion using social media. Although the study was quantitative, it revealed how social media played an important role in health promotion and increased HIV testing uptake among Chinese MSM [23].

In our study, participants’ perceptions on the use of virtual and physical platforms for accessing health-related information and services were explored to determine if and how such platforms could be included in the programme’s engagement strategy.

If the primary focus of an organization targeting MSM and transgender communities is to provide health services, particularly relating to HIV, some beneficiaries may feel that their association with this organisation will lead others to suspect that they are HIV positive. HIV stigma can severely limit MSM and transgender individuals’ social interactions and may further affect their access to health services. We hypothesise that a strategy that includes activities to address discrimination (or links and referrals to such services) may help to disassociate and reduce HIV stigma and ensure that participants are receiving holistic care and support beyond the realm of the biomedical.

Our data revealed that the MSM and transgender communities in Beira create ‘safe spaces’ in places such as bars, barber shops or individual people’s homes, where they can meet, network and socialize. The potential value of such spaces for engagement may not have been adequately utilized for outreach activities involving peer-educators, while formally supported safe spaces have not been tested in this setting. In-depth interviews that were carried out with MSM in Cape Town revealed that Lesbian, Gay, Bisexual, Transgender and Queer (LGBTQ) ‘safe spaces’, even if not always stable and safe, were perceived to be an important part of a strategy for inclusion and emancipation [5].

The involvement of peer educators can be a solution for reaching populations who are highly stigmatised, and may help reach those who have disengaged from health-care services. Their shared experiences and ability to ‘speak the same language’ as their MSM and transgender peers can help build trust, and may assist with improving the recruitment and retention of these communities into health-care services.

This research is subject to a number of important limitations. The focus of the study was to gather information from people who identify as MSM or transgender. As such, it did not seek to explore in-depth how gender, sexuality and social norms in this specific setting affect health seeking behaviour or overall access to care. The narrow scope of the study may have resulted in limited understanding of the experiences of this community in relation to health issues. We are also aware that despite our efforts to recruit “hidden” and “hard to reach” members of the community we are likely to have missed some of the most vulnerable. Our recruitment strategy, through a network of peer-educators, might have resulted in an over-representation of people who were already in contact with other support organisations, NGOs and networks, despite efforts to include those who were not enrolled in MSF’s programme. If this is the case, our findings might underrepresent the challenges that MSM and transgender women face in Beira and similar settings and might have overrepresented the needs and expectations of the more “open” interviewees. Finally, the PI who is part of the MSM and transgender community, works for MSF and is very well known in this context, conducted all the interviews which may have influenced some of the responses.

## Conclusion

Despite these limitations, we believe that our study has elicited important findings pertaining to MSM and transgender communities in a setting heavily affected by HIV and characterized by stigma and discrimination. The current peer-driven model of care was appreciated but could be improved and expanded to take into account the face-to-face and virtual spaces and networks used by interviewees. Whilst social networks are not currently used for the diffusion of health information in this context, this may be an untapped area which could be used to expand services within this highly stigmatised and vulnerable community.

## Supporting information

S1 File Study protocol. (PDF)

S2 File COREQ (COnsolidated criteria for REporting Qualitative research) checklist. (PDF)
