# Supplementary material for: Access to health services for men who have sex with men and transgender women in Beira, Mozambique: A qualitative study
Source: PLoS One. 2020 Jan 30;15(1):e0228307. doi: 10.1371/journal.pone.0228307 (PMC6992190; doi:10.1371/journal.pone.0228307)
Supplement: S1 File — (PDF) [file pone.0228307.s001.pdf]

MINISTÈRIO DA SAÚDE

Study Protocol

WHY ARE MEN WHO HAVE SEX WITH MEN (MSM) IN BEIRA, MOZAMBIQUE, NOT ACCESSING MSM-SPECIFIC SERVICES AND HOW CAN ACCESS TO THESE SERVICES BE IMPROVED?

Principal Investigator/Contact Person: Farisai Gamariel
Operational Research Assistant and Patient Support Supervisor: MSF Beira Project

Email: msfocb-beira-ORAssist@brussels.msf.org
f.gamariel@gmail.com

Phone number: +258 845860613 / 825860610

Version: 14

Date: 15/06/2018

Médecins Sans Frontières OCB – Mozambique– Beira Project

Co-investigators:

Petros Isaakidis: OR Coordinator, SAMU, Cape Town, South Africa:
Petros.Isaakidis@joburg.msf.org

Roles: protocol development, supervision of data collection, data analysis, writing of manuscript

Emilie Venables: Qualitative Research Focal Point, LuxOR/SAMU:
Emilie.VENABLES@brussels.msf.org

Roles: protocol development, supervision of data collection, data analysis, writing of manuscript

Arlete Mahumane: Epidemiologist, CIOB
cynthiwe@icloud.com
Roles: review of protocol and manuscript

Rosita Beula: Central Region Co-ordinator, LAMBDA
miickyfutebol@gmail.com
Roles: review of protocol and manuscript

Vasco Francisco Japissane Cumbe: Research Coordinator – Ministry of Health – Sofala Province:
vcumbe@gmail.com
Roles: review of protocol and manuscript

José Carlos Beirão: Operation Research Manager, Beira Project:
msfocb-Beira-ORmanager@brussels.msf.org
josecarlosbeirao@gmail.com3
Roles: review of protocol and manuscript, support for recruitment and data storage

Ivan Alejandro Pulido Tarquino: Operational Research Coordinator MSFOCB, Maputo:
msfocb-maputo-ORco@brussels.msf.org
Roles: review of protocol and manuscript, support for data collection and data storage, responsibility for local ethics approval

Augusto Manequin: MSFOCB-Beira-Med, Beira Project:
msfocb-beira-Med@brussels.msf.org
Roles: review of protocol and manuscript, support for data collection

Nordino Mulieca: Data Manager, Beira Project:
msfocb-beira-datamanager@brussels.msf.org
Roles: review of protocol and manuscript, support for data storage

Contents

Summary of study ... 6

Abbreviations ... 7

Introduction ... 8

Research Question ... 12

General Objective ... 12

Specific Objectives ... 12

Methodology ... 12

Study design ... 12

Study Population ... 12

Inclusion and Exclusion Criteria ... 13

Recruitment and sampling ... 13

Study period ... 16

Data collection ... 16

In-depth Interview procedures ... 17

Audio-recording ... 18

Transcription and translation ... 18

Data Analysis ... 19

Data storage ... 19

Ethical issues ... 20

MSM in Beira ... 20

Study benefits ... 21

Embarrassment or distress ... 21

Confidentiality ... 22

Informed consent ... 23

Reimbursements ... 23

Policy and practice ... 23

Community engagement ... 24

Expected outcomes ... 24

Dissemination 25
Potential Challenges .... **Error! Bookmark not defined.**
Budget and human resources 26
Bibliography 27
Appendix A: information sheet and informed consent form 30
Appendix B: in-depth interview guide (enrolled men) 35
Appendix C: in-depth interview guide (non-enrolled men) 38

Summary of study

HIV prevalence and incidence are higher among men having sex with men (MSM) in low and middle income countries, in relation to men in the general population (Baral *et al.*, 2007). In the context of the HIV pandemic in Southern Africa, an increasing number of actors, including Médecins Sans Frontières (MSF), are working towards tailor-made services for key populations (including MSM), as recommended by the World Health Organization (WHO 2014). MSF has been providing HIV-related services in Beira, Mozambique, since January 2014, when a project offering HIV prevention and treatment to female sex-workers began. In December 2015 the project started engaging MSM and the first MSM was registered in the programme in January 2016.

This qualitative study will be conducted in Beira with men who have sex with men (MSM). The study aims to understand the health-specific needs and expectations of MSM in Beira, to explore barriers and facilitators in accessing MSM-specific health related services and to identify different strategies and platforms for discussion with and engagement of MSM. The study will ask why MSM in Beira are not accessing MSM-specific services with the ultimate aim of understanding how access to these services could be improved. Engagement platforms could include social media platforms such as Facebook and WhatsApp. Physical platforms could include the identification of places such as bars, barbershops and salons where MSM meet. Identifying these platforms and the kind of services MSM need and expect will enable MSF and collaborators to design specific services to reach MSM in Beira.

A total of 30 in-depth interviews (IDIs) will be carried out with MSM who are 1) enrolled in MSF's programme for MSM in Beira and 2) those who are not enrolled. Participants enrolled in MSF programme will be purposively selected for the study whilst those that are not enrolled will be selected using snowball sampling. Interviews will be conducted in English, Shona or Portuguese and transcribed. Data will be thematically analysed.

The study is expected to take 10 months to complete, from the completion of the protocol and findings will be shared with local partners and disseminated within Mozambique and internationally through presentations and a peer-reviewed publication.

Abbreviations

**AIDS**	Acquired Immunodeficiency Syndrome
**HIV**	Human Immunodeficiency Virus
**HCT**	HIV counselling & testing
**IBBS**	Integrated HIV Bio-Behavioural Survey
**IDI**	In-depth Interviews
**LGBTQ**	Lesbian, Gay, Bisexual, Transgender and Queer
**MSF**	Médecins Sans Frontières
**MSM**	Men who have Sex with Men
**PrEP**	Pre-exposure prophylaxis
**WHO**	World Health Organization

Introduction

HIV prevalence and incidence are higher among men having sex with men (MSM) in low and middle income countries, in relation to men in the general population (Baral *et al.*, 2007, WHO 2014, Sharma A. et al., 2015). In the context of the HIV pandemic in Southern Africa, an increasing number of actors, including Médecins Sans Frontières (MSF), are working towards tailor-made services for key populations (including MSM), as recommended by the World Health Organization (WHO 2014, Sharma A. *et al., 2015*).

Despite the WHO guidelines on providing services to MSM, which recommend using an evidence-based, culturally relevant and rights-based approach, MSM uptake of HIV services in most resource-limited and rights-constrained settings is not as high as expected, often due to stigma. A study conducted in March 2017 in Senegal revealed that even though the country has managed to control HIV and AIDS to prevalence as low as 0.5% in adults of 15 – 49 years, the scenario is different especially in MSM due to stigma. Most of the 172 participants reiterated that stigma was the deterrent factor in accessing health services (Ahanda, 2017).

Just like in Senegal, progress in working with key populations including MSM is still hampered by stigma and discrimination in many contexts. In-depth Interviews (IDIs) that were carried out with MSM in Cape Town, South Africa, revealed that Lesbian, Gay, Bisexual, Transgender and Queer (LGBTQ) 'Safe Spaces' were not always stable and safe but were part of a longer-term political and geographical strategy for inclusion and emancipation (Hassan *et al*, 2018).

Homosexuality has been decriminalized in Mozambique since June 2015. However, stigma and discrimination are still very common (Sjöstrand 2014). Although MSM-friendly, tailor-made health services and packages such as clinics for men, door to door follow up services and after hours clinics are being offered in different communities among key populations, MSM still encounter barriers in accessing these services (Micheni *et al*, 2017). According to Wirtz *et al* (2014), less than 10% of MSM are accessing MSM-specific services globally, and this is even lower in low and middle-income countries. This is particularly worrying as several of those countries,

including Uganda and Gambia, still criminalize homosexuality and "the negative role of stigma in fuelling the HIV epidemic" (Hagopian *et al*, 2017)

In 2011, an Integrated HIV Bio-Behavioral Survey (IBBS) was conducted in three large cities in Mozambique: Maputo, Beira and Nampula (Nala *et al* 2015). The survey aimed to determine the MSM population of these cities, as well as the HIV prevalence among the MSM population. Results showed that 90% of HIV positive MSM who participated in the survey in Mozambique were unaware of their HIV status. In Beira the MSM population was estimated to be 2,624, which was about 1.8% of the total MSM population in Mozambique, and the HIV prevalence among them was 9.1%. More than 90% of all MSM participants regardless of status were unaware of their HIV status. According to the same study, prevalence in Beira was higher among MSM ≥25 compared to those 18-24 years (32.1 vs. 2.8 % respectively), hence the need for prevention strategies for younger MSM and ensure care and treatment for older HIV-infected MSM (Nala *et al* 2015).

Although there are extensive prevention strategies that lower HIV transmission as well as antiretroviral therapy approaches globally, HIV control effectiveness among MSM is limited by structural factors leading to low health-seeking behaviours (Beyrer *et al.,* 2012). However, data for the same communities in low and middle income countries remain extremely limited, non-existent, out of date or inconclusive. There are also limited services for the MSM community (amfAR, 2010). Qualitative evidence may add insights into the experiential dimensions of barriers and facilitators to accessing HIV services (Lorenc *et al.,* 2011) among marginalized communities such as MSM and help to identify factors that may improve access and utilization of such services.

Social media platforms, such as Facebook may give room for those MSM that are fearful or reluctant to be seen accessing health services due to stigma and discrimination to gain information. Social media could be a bridge to access information in health. A study conducted by Boogerd (2015) revealed that social media can be used in different social, economic and cultural contexts, with very short messages being effective in communicating HIV prevention and treatment issues. Social media may also be effective as they offer anonymity and confidentiality to the users. According to Allison (2014), online and offline programmes for MSM engagement

increase the visibility campaigns for health through social networking. These also increase knowledge and help to link health services.

A study carried out among 112 MSM Afro American and Latino participants living in Los Angeles on social media for communication in health concluded that, 'Social networking communities are acceptable and effective tools to increase home-based HIV testing among at-risk populations' (Young, 2013).

Many public health interventions have incorporated social media in their engagement with the MSM community in order to increase adherence to health service access. A study that was carried out in China revealed that health promotion using social media translates to changes in behaviour. Although the study was quantitative, it revealed that social media plays an important role in health promotion and promotes HIV testing among Chinese MSM (Bolin *et al.,* 2017)

The main reason why the study is focusing on social media and physical platforms to engage with MSM is that ANOVA (A South African based organisation working with MSM) conducted a short and rapid assessment in August 2016 when MSF started working with MSM. Their recommendation was that MSF should opt to use social media platforms to engage with MSM to enable the highly stigmatised and discriminated members of the community to access information and will reduce their stigmatisation. Although this study will focus mainly on virtual and physical platforms, other platforms that arise during the interviews will not be ignored.

MSF has been working in Beira since January 2014, when a project offering HIV prevention and treatment to female sex-workers began. In December 2015 the project started engaging MSM as a pilot project: the first MSM was registered in January 2016. When the project began to integrate MSM, the same strategies for outreach as those for the sex-workers were used. A clinic for men, HIV testing and door-to-door follow up services as well as Pre-Exposure Prophylaxis (PrEP) for HIV negative beneficiaries were introduced. In addition, outreach activities are held in the local community. Despite these services, there remains a low uptake of services amongst the MSM population.

The present study aims to understand the health-specific needs and expectations of MSM in Beira, to explore barriers and facilitators in accessing MSM-specific services and to explore different strategies and platforms for engagement with MSM.

Research Questions

> Why are Men Who Have Sex with Men (MSM) in Beira, Mozambique not accessing MSM-specific services and how can access to these services be improved?

General Objective

- To understand why MSM in Beira are not accessing MSM-specific services and to explore how these services can be improved.

Specific Objectives

- To understand the health-specific needs and expectations of MSM in Beira;
- To explore barriers and facilitators in accessing MSM-specific services
- To explore different strategies and/or platforms for engagement with MSM (including in- person and virtual).

Methodology

Study design

This will be a purely qualitative research study using in-depth interviews that will be carried out amongst MSM in Beira, Mozambique. The qualitative study design will allow us to conduct research with a stigmatised and often hard-to-reach population who we know relatively little about. The choice of in-depth interviews as a methodological tool will allow us to discuss potentially sensitive issues in detail, one-on-one with participants rather than in a group setting, which could make some men feel uncomfortable.

Study Population

The study will be carried out with two groups amongst the MSM population in Beira:

1. MSM who are currently registered in MSF’s programme.

2. MSM who are not registered in the programme. These men, who are not registered, may or may not have prior contact with the MSF team.

Inclusion and Exclusion Criteria

Inclusion criteria:

The following are the criteria for both groups of participants to be selected for the study. The participants should be:

a) at least 18 years old;

b) MSM living in Beira at the time of the study;

c) Speakers of Portuguese, Shona or English.

Exclusion criteria:

a) Do not sign the informed consent

Recruitment and sampling

The estimated population of MSM in the city of Beira is 2600 (IBBS Survey, 2011), and approximately 346 MSM were registered at the end of January 2018 in MSF's programme according to MSF's project database in Beira.

A minimum sample of 20 and a maximum sample of 30 participants will be selected for in-depth interviews from the MSM population of Beira, of which up to 15 will be those that are already registered in the MSF program and up to 15 who are not registered in the programme. A previous study by Latham, (2013) suggested that saturation occurs at 11 participants, but with a minimum of 15 for most qualitative studies. On the other hand Crouch and McKenzie (2006) reiterated that up to 20 participants help researchers to maintain close relationship with participants. According to Crouch and McKenzie many qualitative research studies usually require 15 – 20 interview participants, so a suggested sample of 20-30 total participants in the current study might be considered appropriate. IDIs with both groups will be continued until saturation is reached.

Table 1: Maximum participant distribution

Participants	Study activity	Description
10-15	In-depth interviews	Registered with MSF
10-15	In-depth interviews	Not registered with MSF

Table 1 above illustrates how the participants are distributed per group. There will be two sampling procedures included in this study: purposive sampling (among registered MSM) and snowball sampling (among non-registered MSM).

a) **Purposive sampling** will be used to select MSM participants who are currently enrolled in MSF's programme. MSM who are currently registered appear in the MSF database. Their phone numbers will be retrieved by the PI and he will call eligible participants to check their willingness and availability for an interview prior to the interview day. The participant will indicate the interview venue of his choice as well as indicate the most convenient time to them. However those that do not have phone numbers in the MSF database will be contacted through a visit by a peer educator already working for MSF and informed about the study. The peer educators will give a feedback to the PI regarding the participant's willingness to participate. Peer educators will also indicate the time and venue for the interview preferred by the potential participant.

The main reason for having samples of different age ranges is to have wider opinion based on different age categories especially on the platforms of communication. Interviewing participants of different age ranges will also bring in different opinions towards accessing and up taking health services. The reason for selecting a sample of transsexual participants is that they are marginalised and suffer most stigma and discrimination and that they will be able to bring useful information that will help to improve specific health services for the group (Wolf *et al,* 2016*).*

Bisexual men are also important to include as they may access different platforms or networks to other study participants.

Participants selected through purposive sampling may not feel that their confidentiality has been breached since the PI already has a team assisting them in the field and are always informed that MSF team works to preserve their privacy and confidentiality. Once eligible participants will be identified, they will be contacted by the PI by phone. Those who are not reachable by phone will be contacted by the peer educators who are trained to deal will confidentiality issues and will protect participant's privacy. Participants will be also explained that were chosen because they are already MSF clients, and that their identity and any other information will not be shared with anyone.

b) **Snowball sampling** will start from a first participant identified by MSF peer-educators who in turn will identify and invite MSM who are not registered in MSF's programme to participate in this study. Snowball sampling is a technique for finding research participants who may otherwise be hard to recruit. One participant or interviewee gives the researcher the name of someone else they think may want to participate, who in turn provides the name of someone else (Vogt, 1999).

The first participant will be selected from MSM who are not registered in the programme but who has frequent contact with the MSF peer educators. After the interview, this participant will be asked to suggest other MSM from his network who they think may be willing to be interviewed. Each new interviewee will also be asked to suggest other MSM participants and ask them if they would be interested in participating, then whether they prefer to be contacted directly by the PI or to contact the PI themselves. The phone number of the PI will be given to those who prefer to contact the PI themselves. This process will continue until saturation is reached.

For the snowball sampling procedure the PI will ask the participant already identified and already interviewed to suggest another potential participant.

To ensure as wide a range of perspectives as possible, effort will be undertaken during recruitment to ensure that at least two men from the following categories will be included in each group (assuming that these categories will be harder to reach):

- men age 40 and above;
- transgender men;
- bisexual men;

Study period

The whole study will take place within a 12 month period. Once the protocol has been approved the collection of data will take place within a month.

Table 2: Study period

Activity	Period (2018)
**Protocol development including translation of protocol into Portuguese**	*January - February*
**Ethics review (local and MSF)**	*March - June*
**Data collection**	*July-September*
**Transcription and translation/review**	*July-September*
**Data analysis**	*September- November*
**Writing up module**	*November*

Data collection

Prior to data collection, the interview guides will be reviewed and tested with peer-educators. Two pilot interviews will be carried out. After each interview the participants will be asked to

give their views on the questions asked in the interview and give their opinions about the interview procedures. The data collected during this pilot phase will not be used for the analysis.

In-depth Interview procedures

All the interviews will be carried out by the PI, who is fluent in English, Shona and Portuguese. No translator will be required.

The interviews will be held in locations convenient to participants. The participants will be asked to choose the venue that is most convenient to them, for example at their home, their workplace or in the MSF office, where activities for MSM often take place.

The timing of the interviews is also important to consider and the interviewer will be flexible with working hours, based on the timing most convenient to the interviewees (such as conducting interviews in the evenings, if this is preferable to participants).

IDI have been chosen as a data collection tool in order to collect personal, and possibly sensitive data on individual level of understanding of health services and access, views on possible disclosure/stigma, personal beliefs related to the modality and easiness to health care services, experiences with health care services and perceptions towards specific services offered by MSF. Because of the sensitivities surrounding the topic some men may not be comfortable in a group discussion.

It is anticipated that interviews will take between 30 and 60 minutes each. Some consenting participants may be invited to participate in more than one interview sessions, if this is more convenient to them (e.g. if they only have limited time due to work commitments, but want to continue the conversation at a later date). This will also be explained during the informed consent process.

There is a possibility that the questions in the submitted interview guide based on the earlier interviews have wording issues. The PI will carry out two interviews; one with a staff member and another to an MSF MSM beneficiary already in the program to validate the tool. These two

interviews will help to reformulate the questions so that they are accessible to the participants. Please note that these two interviews will not be considered as part of the final data.

Audio-recording

Interview data will be electronically recorded after consent is sought from the participants. If participants do not want to be audio-recorded, verbatim handwritten notes will be relied upon instead. Handwritten notes will be taken during all interviews, if participants agree, but will be the only source of data if audio-recording is not carried out.

Once the IDI is completed, and study participants left the location, the Principal Investigator will review notes made during the IDI and the general atmosphere of the IDI. Important information will be written down on the final page on the Interview guide. Where needed, the formulation of questions will be adapted for the future IDI and probes may be adapted or added. The notes will be considered as part of the collected data.

Transcription and translation

Interviews will be conducted in English, Shona or Portuguese depending on the preference of the interviewee (as mentioned above, the PI is fluent in all three languages). All audio-recordings will be transcribed. If the interview is not audio-recorded, the handwritten notes will be typed up as a transcript. All the transcripts will be in English, Portuguese or Shona depending on which language the interview was done. The Portuguese and Shona transcripts will then be translated into English.

All transcriptions and translations will be conducted by the PI and co-investigators will assist where necessary, for example, to verify any discrepancies in terminology used by interviewees. In order to reduce errors during transcription, the PI will do a weekly quality check. This will reduce the possibility of errors or misunderstanding during transcription. In order also to reduce the recall bias during transcription, the interview will be transcribed during the following day. By doing this, PI also will allow for any possible change in probes or wording of the interview guide, as well checking for saturation of some topics.

Data Analysis

The coding and analysis will be done manually and a thematic approach will be used. This will be done by the PI with support from two co-investigators. All codes will be discussed between the researchers during the process of analysis. We will utilize a thematic network as our framework for analysis, as described by Attride-Stirling, (2001). Basic themes are grouped together with their categories and sub themes. These themes are then reinterpreted to tell a meaningful story. A large text is decoded into smaller but meaningful data that can be easily interpreted.

Data storage

Informed consent forms and paper copies of transcripts will be kept in separate locked cupboards in the MSF office and these will be the responsibility of the Operational Research Manager of the Beira Project who will have the responsibility of keeping the study documents.

The transcripts and other documents will be destroyed after five years, by which time the study will have been completed and published. Audio-recordings will be destroyed after transcription. The principal investigator will be responsible for the destruction of both electronic and paper data files. However, in case the PI is no longer working for MSF, the Operation Research Manager will be responsible for the destruction of the material.

Data verification

The first step in ensuring the quality of the data will happen after the first interview has been conducted. The PI will conduct the interview, translate and transcribe it and send the transcript to two co-investigators for input and review. The same process will happen with the second transcript. Once any challenges with translation and suggestions for improvement to interview techniques made (such as additional areas of probing), the rest of the data collection will continue.

The recruitment strategy of snowballing will also be continuously reviewed and discussed with the research team to see if there are any challenges or ways in which recruitment could be improved.

Field visits by one or two co-investigators may be organized in order to support the PI with the snowballing sampling strategy, interview techniques, data verification and data analysis.

Ethical issues

This study presents some risks. Even after homosexuality has been decriminalised; there is still stigma and discrimination in the society. This implies that there is an enhanced need to protect participants' privacy and confidentiality. In order to reduce unintentional disclosure, the PI together with participant will choose a venue which the participant is comfortable with for the interview. If unexpected problems arise at the venue or the PI or the participant no longer feels at ease, the interview may be stopped or the venue changed. To reduce other possible sources of disclosure the PI will not wear any clothing that identifies MSF.

All members of the research team and peer educators involved in explaining the study to potential participants will receive a focused training about ethics by the Health National Institute, and by the MSF OR Coordinator in Maputo.

This protocol will be submitted to MSF Ethical Review Board and to the Mozambican Comitê Nacional de Bioética para a Saúde (CNBS).

MSM in Beira

Not all MSM in Beira have disclosed their sexual orientation to other people, and many may reluctant to disclose this to others. It may be very difficult to recruit those who have not disclosed their status (those who form part of the 'non-registered' group, and they may be fearful or embarrassed being associated with an organisation such as MSF, as has also been observed in relation to HIV status.

Participants might also not want to be associated with using MSM dating platforms and thus may not want to answer questions relating to these. Participants might also feel uncomfortable to talk about sex and sexual partners.

The experience that the PI has in working with MSM will be an added advantage to the study as this will give an assurance that their involvement in the study, and status as MSM, will remain confidential. In addition, the peer educators working with MSM are very experienced in managing potential issues relating to stigma, and will be able to discuss any challenges with recruitment or stigma with the PI. We are also aware, however, that this could pose a possible challenge as the PI may be seen in the role as 'service provider' working within the programme and not as a researcher conducting interviews and participants may be guarded or uncomfortable in their responses. During the informed consent process, it will be stressed that the research is distinct from the everyday activities of MSF, and that participation will not affect the ability to access services.

Study benefits

The study will not directly benefit participants, but results of the study may, however, help to improve the relevance and quality of the services offered to MSM in Beira, as well as help to improve access to services.

Embarrassment or distress

Due to the nature of the questions, some participants may be embarrassed during the interviews. The PI will explain the purpose of the interview beforehand, and ensure that participants understand why they were selected to participate and that they are free to stop the interview at any time if they do not wish to continue. If any interviewee becomes distressed as a result of the interview, they will be referred to a trained counsellor for further support if they wish.

Confidentiality

Before each interview, the PI will discuss and confidentiality as part of the informed consent process. This is especially important when working with a highly-stigmatised population such as MSM.

Audio recordings will be transferred to a password protected computer belonging to the PI. The recordings will not be shared with anyone else. The PI will transcribe the audio-recordings on this computer and will only share anonymised transcripts with two co-investigators who will be involved in the data analysis.

Whatever information participants provide in the interview will be anonymised at the point of transcription and the participant's identity will not be included in transcripts or any reports, publications or presentations emerging from the data. In addition to the name of the participant, any other names such as those of relatives or friends, will be removed during the transcription process.

During the process of analysis, specific verbatim quotes from participants will be identified and then used in final reports and presentations. If there are any specific details that could identify someone (such as a detailed description of where they work, or their family, for example) these will be removed. Please note that during the transcription and coding participants will not be identified by the co-investigators and the PI will make sure that the participant privacy and confidentiality is protected.

Informed consent forms will be locked in the MSF project office in a dedicated cupboard in which confidential study material for all studies within the project is kept.

The participation of interviewees in the study will not be shared or discussed outside of the research team.

In addition, the PI is collaborating with organisations that have specialised experience and expertise in working with MSM in the Southern African region, thus will be able to provide on-going advice and support in ensuring that individuals cannot be identified.

Informed consent

Verbal informed consent will be taken from all participants before the interview begins. This will show that they agree to be interviewed. Verbal informed consent will also be taken for audio-recordings.

The participant will also be informed that the recorded voice will not be used for broadcasting (eg on the radio or television) but rather for the PI to be able to capture the exact words of the participant while transcribing the data.

If an interviewee withdraws their consent during an interview, the interview will not be transcribed.

Reimbursements

Transport costs will be reimbursed if the participant travels to the interview venue. For this study regardless of distance the amount will be fixed at 50 MT, in line with routine project activities. No other reimbursements will be given for participation in an interview.

Policy and practice

The study will help the project to determine ways to improve specific health packages for MSM. An in-depth understanding of MSM in Beira will help the project to understand any gaps in access and the best platforms to use for communication, and will assist the team to implement suitable activities for the MSM project.

Potential platforms and strategies for engaging with MSM which could be developed may include physical places such as bars, barber shops and salons and virtual ones such as Facebook, Grinder and Whatsapp. These are possible meeting places where MSM may discuss several issues related to them and could be also be conducive to discuss health related issues.

The study findings may also be used to support advocacy efforts for policy change, which could improve the recognition of the specialised needs of MSM in Beira and may be of relevance in other similar settings in Mozambique.

Community engagement

Community engagement is already part of the work done by peer educators within MSF's activities. Care will be taken to ensure that the research activities (interviews) are clearly explained and not confused with routine community engagement activities that take place.

MSF is partnering with other organisations during this study, which will also assist with disseminating the findings to other actors working with MSM in the region.

Expected outcomes

It is expected that the study may help to identify barriers to health services among MSM in Beira and may help to improve and further tailor the services to the community needs. We also expect that the study may reveal the most appropriate in-person and virtual platforms for communication and contact with the MSM community.

There may be some implications in the implementations of the research findings; for instance if the study is halted before saturation is reached a formal report to the MSF and the local ethical review boards will be done. The PI and the co-investigators will sit down to discuss ways to resolve the problem. After the problem has been resolved the study will recommence and the ethical review boards will also be notified.

There are plans to implement the findings and engage local policy makers such as the Ministry of Health and other stakeholders. The study will certainly inform the local policy makers about the work done by MSF. Since in the qualitative design were not included FGDs among participants because of the need of some participants to remain "hidden", more prevalent ideas and concepts about challenges and difficulties in accessing health-related services of MSF, and no particular

key informants were identified, the information collected remain at a very personal level (IDIs) describing personal experience of the interviewed participants. This represents an evident limitation both due to the design of the study and the information achieved. Because of this, findings will be strictly related to the environment of this community in Beira, limiting the generalizability and comparison with other MSM communities.

Dissemination

The study results will firstly be shared with the MSF team through an interactive session to be held in the MSF office. The results of this study will be disseminated to health care workers, local and national authorities as well as international actors and NGOs working within Beira or other parts of the country. The results will also be shared in a report and presented at national and international conferences. Efforts will be made to publish the findings in a peer reviewed journal with a focus on key populations. Feedback will be given to MSM through PI and the network of peer-educators.

Limitations

There are several potential limitations to this study, which will not be known in full until data collection and analysis begin. As MSM in Beira are a highly stigmatised population, and recruiting them to MSF's services has already been challenging, similar challenges in recruitment may occur with the study, thus limiting the scope of the study and causing delays in recruitment.

We also acknowledge the following potential challenges during the recruitment or data collection phase:

- Participants might not want to reveal their MSM status. It is the role of the PI to assure them that their status will remain confidential and not revealed to others;
- Participants might not be aware that MSF is offering specific services to men and thus find it difficult to answer some of the questions or offer their perspectives. Prompts in the interview guide will help to address the problem;

- Participants might not reveal that they are not accessing MSF services because they are already engaged with other organizations offering the same services. Also participants will be assured that their affiliation with MSF will not jeopardize their relationship with other organizations ;
- Participants might highlight that they do not use social networks because they do not have smart phones or computers. The interviewer will also ask questions about physical platforms and will probe about any other ideas the beneficiaries may have;
- Participants might not want to be associated with MSM dating platforms and thus may not want to answer questions relating to this. Participants will also be assured that there is "nothing wrong" in using MSM dating platforms; they will be assured that the PI will keep this information confidential.
- Participants might feel uncomfortable to talk about sex and sexual partners, so it is the responsibility of the PI to assure participants that all their information will be private and confidential.

Budget and human resources

As the study is being conducted as part of MSF's routine activities, no specific budget is required for the study. No additional human resources will be required for the study as it falls under the scope of the PI's work. No additional sources of funding are required for this study.

Bibliography

- Aditi Sharma, Anja Teltschik and Laura Davies. (2015); **HIV and key populations**: Available at: https://www.gnpplus.net/assets/wbb_file_updown/4810/Community%20Guide_I_HIV%20and%20key%20populations.pdf

- Ahanda, K. (2017), Mitigating Stigma in HIV services for Key Populations in Senegal, USAID. Available at https://www.usaid.gov/what-we-do/global-health/hiv-and-aids/success-stories/mitigating-stigma-hiv-services-key-populations

- Allison SM, Adams D, Klindera KC, Poteat T, Wolf RC, (2014). Innovative uses of communication technology for HIV programming for men who have sex with men and transgender persons. J Int AIDS Soc. 2014 Oct 1;17:19041. doi: 10.7448/IAS.17.1.19041. eCollection 2014. PubMed PMID: 25280864; PubMed Central PMCID: PMC4185130.

- amfAR, (2010). MSM and the Global HIV/AIDS Epidemic: Assessing PEPFAR and Looking Forward, Washington, DC. Available at http://www.amfar.org/uploadedFiles/_amfarorg/Around_the_World/IBPepfar(1).pdf

- Attride-Stirling, J. (2001) Thematic network: an analytic tool for qualitative research: London: Sage

- Baral S, Sifakis F, Cleghorn F, Beyrer C. Elevated risk for HIV infection among men who have sex with men in lowand middle-income countries 2000–2006: A systematic review. PLoS Med 2007;4:e339.

- Beyrer C, Baral SD, van Griensven F, Goodreau SM, Chariyalertsak S, Wirtz AL, Brookmeyer R, (2012). Global epidemiology of HIV infection in men who have sex with men. Lancet. 2012 Jul 28;380(9839):367-77. doi: 10.1016/S0140-6736(12)60821-6. Epub 2012 Jul 20. Review. PubMed PMID: 22819660; PubMed Central PMCID: PMC3805037.

- Bolin Cao, Chuncheng Liu, Maya Durvasula, Weiming Tang, Stephen Pan, Adam J Saffer, Chongyi Wei, Joseph D Tucker, (2017); Social Media Engagement and HIV Testing Among Men Who Have Sex With Men in China: A Nationwide Cross-Sectional Survey J Med Internet Res. 2017 Jul; 19(7): e251. Published online 2017 Jul 19. doi: 10.2196/jmir.7251 PMCID: PMC5541240

- Boogerd EA, Arts T, Engelen LJ, van de Belt TH. (2015). "What Is eHealth": Time for An Update? JMIR Res Protoc. 2015 Mar 12;4(1):e29. doi: 10.2196/resprot.4065. PubMed PMID: 25768939; PubMed Central PMCID: PMC4376129.

- Crouch, M., & McKenzie, H. (2006). The logic of small samples in interview-based qualitative research. *Social Science Information, 45*(4), 18. doi: 10.1177/0539018406069584

- Gunther Eysenbach. (2015); Social Media and HIV: A Systematic Review of Uses of Social Media in HIV Communication, Bethesda MD, 20894 USA, Available at https://www.ncbi.nlm.nih.gov/pmc/articles/PMC4642795/

- Hagopian A, Rao D, Katz A, Sanford S, Barnhart S. (2017), Anti-homosexual legislation and HIV-related stigma in African nations: what has been the role of PEPFAR? Glob Health Action; 10(1):1306391. doi: 10.1080/16549716.2017.1306391. PubMed PMID: 28580879; PubMed Central PMCID: PMC5496073.

- Hassan NR, Swartz L, Kagee A, De Wet A, Lesch A, Kafaar Z, Newman PA. "There is not a safe space where they can find themselves to be free": (Un)safe spaces and the promotion of queer visibilities among township males who have sex with males (MSM) in Cape Town, South Africa. Health Place. 2018 Jan;49:93-100. doi: 10.1016/j.healthplace.2017.11.010. Epub 2017 Dec 9. PubMed PMID: 29227887.

- Lorenc T, Marrero-Guillamón I, Llewellyn A, Aggleton P, Cooper C, Lehmann A,Lindsay C. HIV testing among men who have sex with men (MSM): systematic review of qualitative evidence. Health Educ Res. 2011 Oct;26(5):834-46.

- Micheni M, Kombo BK, Secor A, Simoni JM, Operario D, van der Elst EM, Mugo P, Kanungi J, Sanders EJ, Graham SM. Health Provider Views on Improving Antiretroviral Therapy Adherence Among Men Who Have Sex with Men in Coastal Kenya. AIDS Patient Care STDS. 2017 Mar;31(3):113-121. doi:10.1089/apc.2016.0213. Epub 2017 Feb 22. PubMed PMID: 28282249; PubMed Central PMCID: PMC5359680.

- Latham, J. R. (2013). A framework for leading the transformation to performance excellence part I: CEO perspectives on forces, facilitators, and strategic leadership systems. *Quality Management Journal, 20*(2), 22. | Download

  Nalá R, Cummings B, Horth R, Inguane C, Benedetti M, Chissano M, Sathane I, Young P, da Silva D, Mirjahangir J, Grasso M, Fisher Raymond H, McFarland W, Lane T.(2015). Men who have sex with men in Mozambique: identifying a hidden population at high-risk for HIV. AIDS Behav;19(2):393-404. doi: 10.1007/s10461-014-0895-8. PubMed PMID: 25234252; PubMed Central PMCID:PMC4341016.

- PEPFAR, (2017); Mozambique Operational Plan (COP/ROP) 2017; Strategic Direction: Available at https://mz.usembassy.gov/wp-content/uploads/sites/182/2017/04/SBU_Moz-SDS-2017_3.02.17-SUBMITTED-v2-PUBLIC-1.pdf

- Sjöstrand S, (2014); Homosexuality decriminalized in Mozambique; Available at: http://afrikagrupperna.se/jamstalldhet/homosexualitet-lagligt-i-mocambique/

- Vogt, W. P. (1999), *Dictionary of Statistics and Methodology: A Nontechnical Guide for the Social Sciences*, London: Sage.

- Wirtz AL, Kamba D, Jumbe V, Trapence G, Gubin R, Umar E, Strömdahl SK, Beyrer C, Baral SD, (2014). A qualitative assessment of health seeking practices among and provision practices for men who have sex with men in Malawi. BMC Int Health Hum Rights. 2014 Jun 3;14:20. doi: 10.1186/1472-698X-14-20. PubMed PMID: 24893654; PubMed Central PMCID: PMC4049421.

- Wolf RC, Adams D, Dayton R, Verster A, Wong J, Romero M, Mazin R, Settle E, Sladden T, Keatley J, (2016). Putting the t in tools: a roadmap for implementation of new global and regional transgender guidance. J Int AIDS Soc. 2016 Jul 17;19(3 Suppl 2):20801. doi: 10.7448/IAS.19.3.20801. eCollection 2016. PubMed PMID: 27431472; PubMed Central PMCID: PMC4949319.

- Young SD, Cumberland WG, Lee SJ, Jaganath D, Szekeres G, Coates T, (2013). Social networking technologies as an emerging tool for HIV prevention: a cluster randomized trial. Ann Intern Med. 2013 Sep 3;159(5):318-24. doi: 10.7326/0003-4819-159-5-201309030-00005. PubMed PMID: 24026317; PubMed Central PMCID: PMC3879120.

Appendix A: information sheet and informed consent form

Title of study: Why are Men Who Have Sex with Men (MSM) in Beira, Mozambique not accessing MSM-specific services and how can access to these services be improved?"

Name of Principal Investigator: *Farisai Gamariel, Tel: +258 845860613 / 825860610*

Here is some information to help you decide whether you want to participate in this study about why MSM in Beira, Mozambique, do not access specific services and how these services could be improved.

The objectives of this study are:

- To understand the health-specific needs and expectations of MSM in Beira
- To explore barriers and facilitators in access to MSM-specific services in order to improve access and uptake of these services;
- To access different strategies and / or platforms for engagement with MSM (including in person and virtual)

You are encouraged to ask questions or raise concerns at any time about the nature of the study or the methods being used.

Who is conducting this study?

Médecins Sans Frontières (MSF) are conducting these interviews in Beira to learn more about the reasons why men do not access MSF's services. MSF is an international, independent, medical humanitarian organisation that delivers emergency aid to people affected by armed conflict, epidemics, natural disasters and exclusion from healthcare. MSF offers assistance to people based on need, irrespective of race, religion, gender or political affiliation. MSF started working in Mozambique for some time and in Beira it started working in 2014.

Now MSF is conducting this study to help improve our services and make them more accessible and relevant to men in Beira. MSF will conduct interviews with MSM for this study.

The interview will take place in a location of your choice. You will be interviewed only once. This interview will take about 30 – 60 minutes interview about 30 men in total. We are going to interview between 20 and 30 men in total.

Audio recording and notes

Our discussion will be audio-recorded to help me accurately capture what you are saying word for word. The recordings will only be heard by me for the purpose of the study. If you feel uncomfortable with the recorder, you may ask that it be turned off at any time. Handwritten notes will be taken during the interview as a back-up to the recording, or as an alternative if you do not want to be recorded. We will translate the interviews into English afterwards if the interview is in Shona or Portuguese.

Study withdrawal

You also have the right to withdraw from the study any time. In the event you choose to withdraw from the study all information you provide (including audio-recording) will be destroyed and omitted from the analysis and any publications or presentations emerging from the study. Your decision to participate or withdraw will not affect your access to MSF's services now or in the future.

Risks and benefits

There will be no direct benefit to you if you decide to participate in this study. Your involvement may help us to improve our programmes and services for men, which could help other men in your community in the future.

You might feel embarrassed about some of the questions that we ask you but we will minimise this discomfort as much as possible. If you become distressed or upset during the interview and would like to talk to one of our counselling team, this can be arranged.

Although we will not share your name or data with anyone else, someone might see you during the interview and learn about your participation in the study. If they know what the study is about, you may feel embarrassed.

We will remove your name from all of the documents emerging from the study so that you remain confidential. Although direct quotes from you may appear in the report, your name and other identifying information will be kept confidential.

Reimbursements

You will not be reimbursed for your participation in the study, but we will pay **50 MT** for your transport fare if you travel to meet us.

Dissemination

Information from you and other participants will be used in reports, presentations for conferences within Mozambique and internationally, and publications in a peer-reviewed scientific journal, which will be presented by myself or another member of the research team. This will all be anonymous: your name will not appear anywhere. Please be advised that your privacy and confidentiality is of great importance to us. Your identity will be protected and that your name will not appear anywhere.

Ethical approval

This study has been approved by the MSF Ethical Review Board and the Comitê Nacional de Bioética para a Saúde (CNBS) [add dates and references once approved].

Further information

If you have any questions want more information about the rights as a research participant or complaints please contact the CNBS on + 258 82–406–6350. The study reference number is [add study number]. You can also contact myself, Farisai Gamariel, the PI of the study if you have any questions or wanting to know more about the study on the following: + 258 845860613 / 825860613

Informed Consent Form (verbal)

Participant understands that taking part in the study is his/her choice and that s/he can decide not to take part. S/he understands that s/he can change his/her mind about participating at any point in the study, and can also stop the interview at any time.	
Participant agrees to being audio-recorded.	

Name of researcher taking consent	
Signature of research taking consent	
Date	

Appendix B: in-depth interview guide (enrolled men)

Thank you for agreeing to participate in this interview today. I am very interested in hearing your views about the services currently provided by MSF in Beira. Your opinion is important and there are no right or wrong answers. You are free to stop the interview at any time if you do not want to continue and you can ask questions at any time.

Obtained verbal informed consent for interview and audio record ☐

MSF and Services

1. Can you tell me what you know about MSF and the services MSF provides in Beira?
Prompts: *knowledge of organisation / health services / HIV counselling and testing (HCT)/ART*

2. Can you tell me, if there is anything, that you know about MSF's services specifically for MSM?
Prompts: understanding of services, understanding of need for MSM specific services

3. Can you tell me about any services you have received from MSF?
Prompts: *HCT/ STI/ Condoms, Gel/lubricant, information*

4. Can you tell me about any additional services you wish to receive through MSF?
Prompts: *social support, emotional support, information on sexual practices*

5. Have you ever spoken to one of MSF's peer educators? Can you tell me about this relationship?
Prompts: *where met the peer educator, how often they see a peer educator, feeling about home visits*

6. Why did you decide to enrol in MSF's programme for MSM? What do you think are the benefits of the programme for you?
Prompts: *relevance of services, did not understand the programme when I joined*

For MSM who are enrolled in the programme, but not active:

7. Can you tell me why you enrolled in the programme, but have not attended any events/accessed any services?
Prompts: *timing & location, convenience, transport, disclosure/did not want to be seen, fear of stigmatisation, felt pressured to join /had been to a previous event and did not find it useful*

8. If you did attend an event, can you tell me more about it?
Prompts: *Debate / film shows / theatre plays / mobile clinic / health fair etc.*

9. Can you tell me about services you wish to receive through MSF that would make you to join?
Prompts: *social support, emotional support, information on sexual practices*

Platforms for communication

We would like to learn more about how MSM communicate with others, to help us improve our programmes and communication with our beneficiaries. I would like to ask you some questions about your networks with friends, and any social networks you might use.

10. How do you arrange to meet with friends in Beira? How do you meet new people? How do you meet new sexual partners?
Prompts: *ask for detailed description of places and communication process*

11. Where in Beira do you often meet to talk with your friends?
Prompts: *Venues? Barber shops, bars. [Ask for specific names of places, as well as times when they are most popular/crowded]*

12. What topics do you often discuss at these meeting places?
Prompts: *Social issues / about other men/ about sex / about health / about business etc.*

13. Do you ever access the internet? Can you tell me more about how you do this?
Prompts: *Smartphone/tablet/laptop? Internet café? Work computer?*

14. Do you ever face difficulties in accessing internet?
Prompts: *Poor network, high data costs, lack of 3G phone, computer etc*

15. Do you use social media? What do you use it for?
Prompts: *FaceBook/Whatsapp/Instagram/snapchat or others*
MSM dating sites and apps: *Tinder/Grindr/Planet Romeo/Scruff or others*

16. What topics do you often discuss using social networks?

Prompts: *Social issues / about other men / about sex / about health / about business etc.*

17. How would you feel about receiving health messages, sexual health information and services that are offered to men through social media? Do you have concerns about confidentiality, and can you explain why/why not?

Thank you for your participation. Do you have any comments about the interview or any questions for me?

Appendix C: in-depth interview guide (non-enrolled men)

Thank you for agreeing to participate in this interview today. I am very interested in hearing your views about services currently provided by MSF in Beira. Your opinion is important and there are no right or wrong answers. You can stop the interview at any time and ask questions if you would like.

Obtained verbal informed consent for interview and audio record ☐

MSF and Services

1. Can you tell me what, if anything, you know about MSF and the services MSF provides in Beira?
Prompts: *knowledge of organisation / health services / HIV counselling and testing*

2. Can you tell me if there is anything that you know about MSF's services specifically for MSM?

3. Can you tell me about any services you may have received from MSF at any time? If any, how was the experience?
Prompts: *talked to a peer educator, got condoms/lubricant, HCT, attended an event*

4. You are not currently enrolled in our programme. Can you tell me why you did not enrol?
Prompts: *did not know about the programme, did not think it was relevant, did not identify with it*

5. Is there anything we could do to make our services more attractive to you?
Probes: *change of venue, kind of activity, information given, timing of services*

Platforms for Communication

We are interested in learning more about how men communicate, to help us improve our services. I would like to ask you some questions about your networks with friends, and any social networks you might use.

6. How do you arrange to meet with friends in Beira? How do you meet new people? How do you meet new sexual partners?
Prompts: *ask for detailed description of places and communication process*

7. Where in Beira do you often meet to talk with your friends?
Prompts: *Venues? Barber shops, bars. [Ask for specific names of places, as well as times when they are most popular/crowded]*

8. What topics do you often discuss at these meeting places?
Prompts: *Social issues / about other men/ about sex / about health / about business etc.*

9. Do you ever access the internet? Can you tell me more about how you do this?
Prompts: *Smartphone/tablet/laptop? Internet café? Work computer?*

10. Do you ever face difficulties in accessing internet?
Prompts: *Poor network, high data costs, lack of 3G phone, computer etc*

11. Do you use social media? What do you use it for?
Prompts: *FaceBook/Whatsapp/Instagram/snapchat or others*
MSM dating sites and apps: *Tinder/Grindr/Planet Romeo/Scruff or others*

12. What topics do you often discuss using social networks?
Prompts: *Social issues / about other men / about sex / about health / about business etc*

13. How would you feel about receiving health messages, sexual health information and services that are offered to men through social media? Do you have concerns about confidentiality, and can you explain why/why not?

Thank you for your participation.
Do you have any comments about the interview or any questions for me?
